# Agrimol B inhibits colon carcinoma progression by blocking mitochondrial function through the PGC-1α/NRF1/TFAM signaling pathway

**DOI:** 10.3389/fonc.2022.1055126

**Published:** 2022-12-14

**Authors:** Dongyang Xiang, Wenjuan Yang, Zihan Fang, Jialei Mao, Qiuying Yan, Liu Li, Jiani Tan, Chengtao Yu, Jun Qian, Dongxin Tang, Xiaoting Pan, Haibo Cheng, Dongdong Sun

**Affiliations:** ^1^ College of Pharmacy, Guizhou University of Traditional Chinese Medicine, Guiyang, China; ^2^ Jiangsu Collaborative Innovation Center of Traditional Chinese Medicine Prevention and Treatment of Tumor, Nanjing University of Chinese Medicine, Nanjing, China; ^3^ Oncology Department, Kunshan Hospital Affiliated to Nanjing University of Chinese Medicine, Kunshan, China; ^4^ The First Clinical Medical College, Nanjing University of Chinese Medicine, Nanjing, China; ^5^ Department of Oncology, Affiliated Hospital of Nanjing University of Chinese Medicine, Nanjing, China; ^6^ School of Integrated Chinese and Western Medicine, Nanjing University of Chinese Medicine, Nanjing, China

**Keywords:** agrimol B, colorectal cancer, PGC-1α, ROS, mitochondrial activity

## Abstract

**Background:**

The activation of peroxisome proliferator-activated receptor-γ coactivator 1α (PGC-1α) stimulates the transcription of the downstream target proteins, mitochondrial transcription factor A (TFAM) and nuclear respiratory factor 1 (NRF1), which induces mitochondrial biogenesis and promotes colorectal tumorigenesis. Agrimol B (Agr) is a constituent of *Agrimonia pilosa* Ledeb. that exerts anticancer effects. Herein, we aimed to investigate the antitumor activity of Agr and its mechanism of action.

**Methods:**

The interaction between Agr and PGC-1α was predicted by molecular docking. After the treatment with different concentrations of Agr (0, 144, 288, and 576 nM), the cell viability, migration rate, proliferation rate, and apoptosis rate of human colon cancer HCT116 cells were determined. Mitochondrial activity, cellular reactive oxygen species (ROS), and mitochondrial membrane potential were assessed to measure the regulatory effect of Agr on mitochondrial function. Western blotting (WB) assay was used to examine the expression of PGC-1α, NRF1, and TFAM, as well as of the pro-apoptotic proteins, Bax and Caspase-3, and the antiapoptotic protein (Bcl-2). Finally, subcutaneous tumor xenograft model mice were used to evaluate the effect of Agr on colorectal cancer (CRC) *in vivo*.

**Results:**

The molecular docking results revealed a high likelihood of Agr interacting with PGC-1α. Agr inhibited the proliferation and migration of HCT116 cells, promoted ROS production and mitochondrial oxidative stress, inhibited mitochondrial activity, and decreased mitochondrial membrane potential. Agr induced cell apoptosis and, in combination with PGC-1α, impaired mitochondrial biogenesis and suppressed the expression of NRF1 and TFAM. Agr also suppressed the expression of Bcl-2 and Cleaved-Caspase-3 and increased the expression of Bax and Caspase-3. In addition, the *in vivo* antitumor effect and mechanism of Agr were confirmed by using a subcutaneous tumor xenograft mouse model.

**Conclusions:**

Our findings demonstrated that Agr regulates the expression of PGC-1α, thereby inducing mitochondrial dysfunction and promoting tumor cell apoptosis. This work highlights the potential of Agr as a promising therapeutic candidate in CRC.

## Introduction

Colorectal cancer (CRC) is the most prevalent digestive system tumor, ranking the third highest and second highest in terms of morbidity and mortality, respectively. CRC is an aggressive and fatal malignancy that has resulted in >160,000 deaths in China (1). Multiple drugs, including cetuximab, panitumumab, bevacizumab, regorafenib, aflibercept, and 5-fluorouracil (5-FU), have extended the overall survival of CRC patients, but fewer than 20% of the patients survive beyond 5 years post-diagnosis due to cancer metastasis and drug resistance (2, 3). Therefore, the development of new drugs and the exploration of their pharmacological mechanisms are crucial for improving the treatment outcomes of CRC.

Peroxisome proliferator-activated receptor-γ coactivator 1α (PGC-1α) is a member of the PGC-1 family of nuclear hormone receptor coactivators. PGC-1α induces mitochondrial biogenesis by stimulating reactive oxygen species (ROS) accumulation (3, 4). Tumor development is closely associated with mitochondrial biogenesis. Clinical studies have indicated that elevated PGC-1α expression accelerates tumor growth, whereas the depletion of PGC-1α is linked to carcinostatic functions (5).

Mitochondrial biogenesis is stimulated by nuclear respiratory factor 1 (NRF1) and mitochondrial transcription factor A (TFAM), which are the downstream molecules of PGC-1α. NRF1 is an essential, intermediate transcription factor in mitochondrial biogenesis, while the activation of TFAM depends on the NRF1-mediated transcription of mitochondrial DNA. Demethylation of *PGC-1α* reduces its inhibition of NRF1 and TFAM expression, resulting in mitochondrial dysfunction and apoptosis (6).

*Agrimonia pilosa* Ledeb., a traditional Chinese herb, possesses anti-inflammatory, antioxidant, and antinociceptive effects (7–9). Agrimophol (agrimol A, B, and C), the main constituent of *A. pilosa* Ledeb., has been widely investigated for the treatment of osteosarcoma, pancreatic carcinoma, prostatic carcinoma, and lung carcinoma (10–12). The correlation between an abnormal increase in mitochondrial membrane potential and poor therapeutic outcomes in CRC has been reported (13). Agrimoniin has been shown to increase ROS content and further decrease mitochondrial membrane potential, resulting in antitumor activity (10). Mitochondrial biogenesis depends on normal mitochondrial membrane potential to control cell apoptosis.

Thus, we hypothesized that agrimol B might prevent the progression of CRC by inhibiting PGC-1α/NRF1/TFAM signal transduction. This study will provide useful insights into i) the molecular mechanism of agrimol B and ii) the antineoplastic activity of agrimophol *in vivo* and *in vitro*.

## Methods

### Molecular docking

The PGC-1α binding pocket was visualized using the AlphaFold Protein Structure Database, https://www.alphafold.ebi.ac.uk/ , followed by the DS molecular docking software to simulate calculating binding sites. Potential targets were stored in PDB (https://www.rcsb.org/) format (ID: AF-Q9UBK2-F1), and the 3D structure of Agr was identified in the PubChem database (CAS: 55576-66-4). The precise docking of the CDOCKER docking module was performed using Discovery Studio 2016 to obtain the binding energy between the receptor and protein. Then, the non-bonding interactions between PGC-1α amino acids and Agr were identified through the interaction analysis between the molecule and the protein, and a two-dimensional diagram of interactions was generated. The same assay was used for the PPARγ protein (ID: AF-P37231-F1).

### Microscale thermophoresis

The interaction between Agr and PGC-1α was measured using microscale thermophoresis with the NanoTemper Monolith NT.115 instrument set at 2% Pico-RED and Medium MST power. Each measurement consisted of 16 reaction mixtures wherein the concentration of fluorescently labeled PGC-1α was set at 20 nM, and twofold dilutions of Agr ranging from 16,000 to 0.488 nM were prepared. The MO.affinity Analysis v2.3 software was used to fit the data and determine the *K_D_* value.

### Cell viability assay

**Figure 1:**
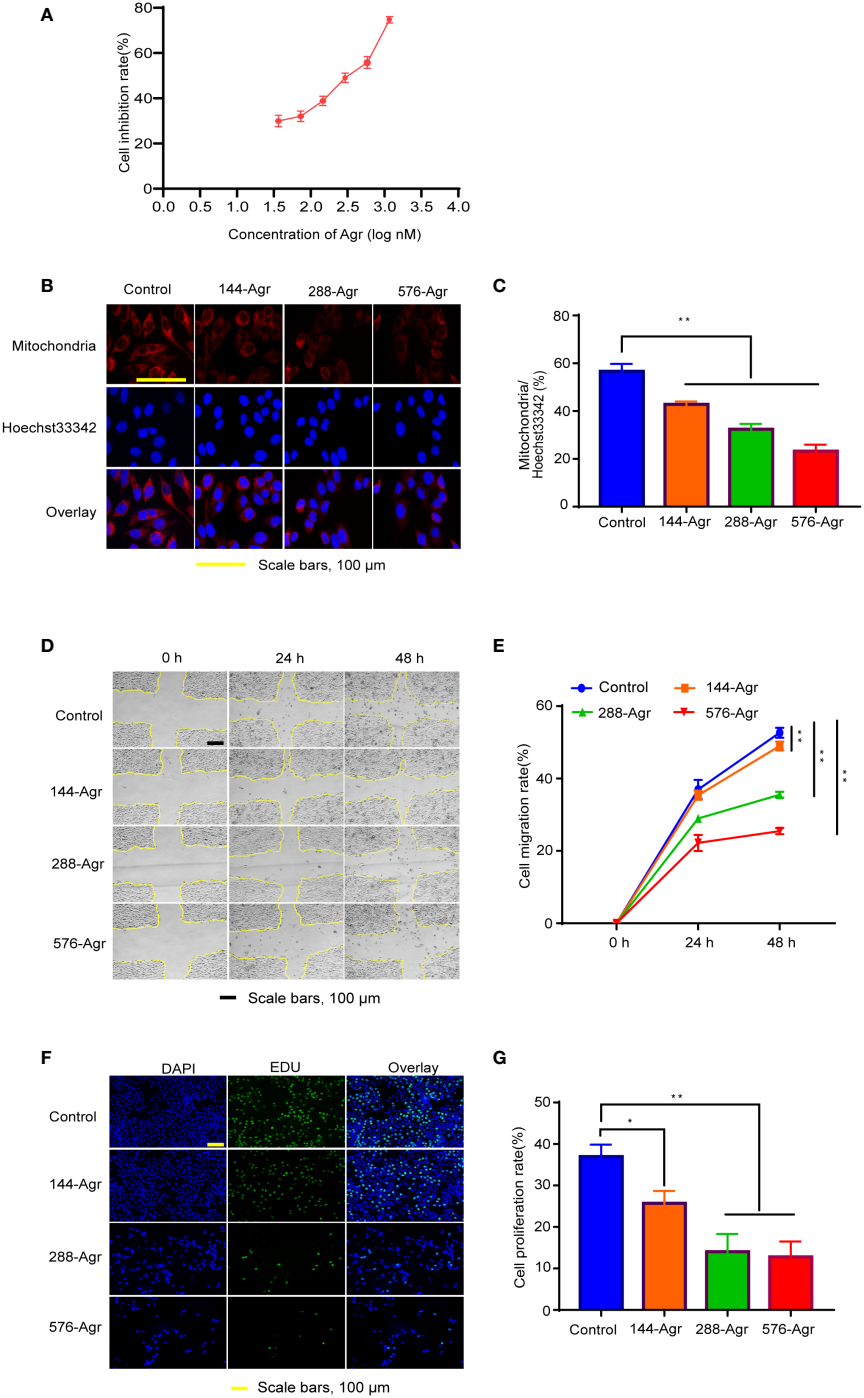
Agr inhibits HCT116 cell proliferation and invasion by modulating mitochondrial functions. **(A)** Cytotoxicity of Agr (36, 72, 144, 288, 576, and 1,152 nM) against HCT116 cells using the MTT assay. **(B)** Confocal fluorescence images of HCT116 cells with mitochondrion/Hoechst 33342 double staining. Cell mitochondria stained with MitoTracker Deep Red FM showed a red fluorescence signal; Hoechst 33342-labeled nuclei showed a blue fluorescence signal. **(C)** The proportion of cells with mitochondrial dysfunction in HCT116 cells after treatment with different Agr concentrations. **(D)** Representative photographs of the wound-healing assay. **(E)** HCT116 cell migration rate after treatment with different Agr concentrations. **(F)** The effects of Agr on HCT116 cell proliferation were measured *via* EdU staining; nuclei in blue (DAPI) and daughter cells in green (EDU). **(G)** HCT116 cell proliferation rate after different Agr treatments. The data processing was shown as mean ± SD (*n* = 3, each group). **p < 0.01, *p < 0.05 *vs.* control. Scale bars = 100 μm. Agr, agrimol B; PGC-1α, peroxisome proliferator-activated receptor-γ coactivator 1α.

The (3-(4,5-dimethylthiazol-2-yl)-2,5-diphenyltetrazolium bromide) MTT assay was performed to determine the effective concentrations of Agr (Macklin, A823603, Shanghai, China). Various concentrations of Agr (0, 36, 72, 144, 288, 576, and 1,152 nM) solutions were prepared in Dulbecco’s modified Eagle medium (DMEM) (Gibco, 11965092, Grand Island, NY, USA) and filtered through 0.22-μm membrane filters. HCT116 cells (iCell Bioscience Inc., HDCL-025, Shanghai, China) were seeded in 96-well plates, and the cells were exposed to Agr at the aforementioned concentrations for 24 h. MTT (Biosharp, 201108, Hefei, China) was used to assess the cell viability following the instrument. The reduction in cell viability was calculated as 1 – OD_control_/OD_Agr_. The IC_50_ value was found to be 280 nM using analysis software (**Figure 1A**). Therefore, the concentrations of *144, 288, and 576 nM* of Agr were selected for subsequent experiments.

### Mitochondrial activity assay

Mitochondrion/Hoechst 33342 double staining was used to estimate mitochondrial activity. HCT116 cells were cultured in six-well plates and exposed to Agr for 24 h. After the 24-h incubation, MitoTracker Red CMXRos (Beyotime Biotechnology, C1049B, Shanghai, China) and Hoechst 33342 (Beyotime Biotechnology, C1025, Shanghai, China) were added to the culture medium. After an incubation period of 20 min at 37°C in a CO_2_ incubator, mitochondrial morphology was observed by confocal microscopy using ImageJ software (NIH, Bethesda, MD, USA) for calculating mitochondrial content.

### Wound-healing assay

The inhibitory effects of Agr on the migration of HCT116 cells were determined using a wound-healing assay as previously described (14). A sterile pipette tip was used to scratch HCT116 cell monolayers, and the wounded monolayers were incubated with Agr (144, 288, and 576 nM) and imaged at 0, 24, and 48 h. Finally, ImageJ software was used to calculate cell migration rates.

### EdU staining assay

5-Ethynyl-2′-deoxyuridine (Edu) was used to assess whether Agr inhibited the proliferation of HCT116 cells. HCT116 cells were seeded onto 48-well plates for 24 h and treated with different concentrations of Agr (0, 144, 288, and 576 nM). The Edu staining was carried out in accordance with the manufacturer’s instructions (UElandy, C6015S, Suzhou, China). The fluorescence intensity was observed under an inverted fluorescence microscope (Olympus Corporation, IX73-A21PH, Tokyo, Japan).

### Mitochondrial membrane potential assay

HCT116 cells were treated with different concentrations of Agr for 24 h, harvested, and treated with 1 μg/L of JC-1 (UElandy, J6004, Suzhou, China) in the dark for 15 min. Next, the cells were rinsed in phosphate-buffered saline (PBS; Solarbio, KGB500, Beijing, China) and suspended with 1 ml of PBS buffer. Finally, the mitochondrial membrane potential was analyzed by flow cytometry, and the results were obtained using ImageJ software.

### *Annexin V-*fluorescein isothiocyanate*/propidium iodide staining assay*

To examine apoptosis, HCT116 cells were treated with different Agr concentrations and trypsinized using trypsin without EDTA (Beyotime Biotechnology, KGY001). The cell suspensions were centrifuged at 300 × *g* for 5 min at 4°C, and the pellets were suspended with Annexin V binding buffer (UElandy, Y6002, Suzhou, China). The suspensions were incubated for 10 min with Annexin V–fluorescein isothiocyanate (FITC) and propidium iodide (PI) staining solution (5 μl:10 μl), and the distribution of the fluorophores was recorded using flow cytometry and measured with ImageJ software.

### Cellular reactive oxygen species detection assay

After HCT116 cells were treated with different concentrations of Agr for 24 h, they were treated with the DCFDA-ROS assay solution (Nanjing Jiangcheng Bioengineering Institute, Nanjing, China; 10 μM) and incubated in the dark. Finally, the intracellular levels of ROS were measured using flow cytometry, and the results were measured using ImageJ software.

### Cell cycle analysis

The cell cycle kit (UElandy, C6031, Suzhou, China) was used for measuring cell cycle effects. HCT116 cells (1 × 10^6^) were incubated with 70% ethanol at 4°C for 12 h and labeled with a PI staining solution in the dark for 0.5 h. The cells were analyzed using flow cytometry, and the segments of layover in the G, S, and M phases were estimated using ImageJ software.

### Animal treatment

Subcutaneous tumors were established by implanting 1 × 10^6^ HCT116 cells into 36 male, BALB/c nude mice as described by Hagiwara et al. (15). When the diameters of the tumors were ≥5 mm, the 35-day-old nude mice (SPF (Beijing) Biotechnology Co., Ltd., SCXK2019-0010, Beijing, China) were randomized into the following four groups: a low-dose Agr group (10-Agr, *n* = 9), which received 10 mg/(kg·day) Agr; a high-dose Agr group (20-Agr, *n* = 9), which received 20 mg/(kg·day) Agr; a control group (control group, *n* = 9), which received PBS; and a positive control group, which received an intraperitoneal injection of 5 mg/(kg·day) 5-FU (Macklin, F80934, Shanghai, China). All groups were injected intraperitoneally once daily for 21 consecutive days. The maximum length and width of the tumor size were measured every 3 days by using digital Vernier calipers (Biao Kang, SL01-22, Shenzhen, China). The tumor volume was calculated by using the formula: length × (width)^2^. Following the administration of the last dose, all animals were euthanized by using ether anesthesia, and then the tumors were excised and weighed under aseptic conditions. After the tumors were washed with PBS, the samples were used for hematoxylin and eosin (H&E) staining, immunofluorescence, and Western blotting. The animal experiment protocol complied with international guidelines and was also approved by the Nanjing University of Chinese Medicine (No. 202204A033).

### Hematoxylin and eosin staining

H&E staining was carried out by using the Hematoxylin and Eosin Staining Kit (Solarbio, Cat# G1120, Beijing, China). In brief, tumor tissues were embedded in paraffin, dehydrated, dewaxed, sliced into sections, and stained. Next, the sections were observed under a microscope and photographed.

### Cell proliferation-associated nuclear antigen Ki-67 assay

Ki-67 is a nuclear marker protein, which is abundantly expressed in sub-G2 and sub-M CRC cell populations. Tumor tissues were treated with 4% paraformaldehyde for fixation, washed in PBS three times, and blocked in 1% bovine serum albumin (Solarbio, A8020, Beijing, China) for 1 h. The tissues were then incubated with primary antibodies against Ki-67 (Cell Signaling Technology, ab15580, Boston, MA, USA) at 37°C for 2 h, followed by secondary antibodies (Affinity Biosciences, S0001, Cincinnati, OH, USA) against proliferating cell nuclear antigen (PCNA) for 1 h at 4°C away from light. The nuclei were stained blue with 4′,6-diamidino-2-phenylindole (DAPI, Solarbio, C0065, Beijing, China), the samples were observed under a microscope, and the results were analyzed using ImageJ software.

### Western blotting analysis

Total protein extracts were obtained from the tumor tissues. Antibodies against GAPDH (Affinity, AF7021, Cincinnati, OH, USA), Cleaved-Caspase-3 (Abcam, ab49822, Cambridge, UK), PGC-1α (9662, Boston, MA, USA), NRF1 (46743, Boston, MA, USA), TFAM (8076, Boston, MA, USA), Bcl-2 (15071, Boston, MA, USA), Bax (5023, USA), and Caspase-3 (9662, Boston, MA, USA) were purchased from Cell Signaling Technology to estimate the respective protein levels as described previously [16]. The expression of the target proteins was calculated using GAPDH as the internal reference.

### Cleaved-Caspase-3 activity assay

Enhanced chemiluminescence (ECL) was used as a readout in the Cleaved-Caspase-3 activity assay. In brief, in the last step of Western blotting (WB) of Cleaved-Caspase-3, ECL fluids (Affinity Biosciences, K002, Cincinnati, OH, USA) were added, and the polyvinylidene difluoride (PVDF) membrane can be covered. The nuclear was stained blue with DAPI. The samples were observed under a microscope and analyzed using ImageJ software.

### Statistical analyses

For all experiments, the original data were analyzed using GraphPad Prism Version 8.0.2 (USA). All data were presented as mean ± SD. One-way ANOVA was used to compare the groups; a *p*-value <0.05 was considered statistically significant.

## Results

### Agr may bind with the active site of PGC-1α

**Figure 2:**
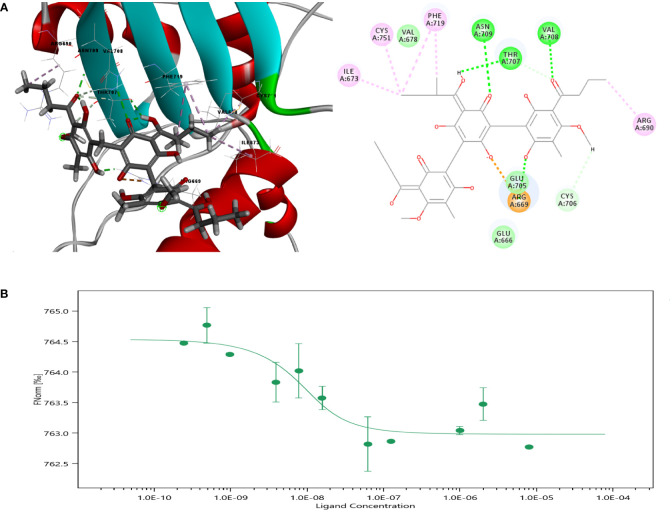
Molecular docking of Agr with PGC-1α. **(A)** Crystal structure of PGC-1α and amino acid sites of interactions of Agr with PGC-1α. **(B)** Results of microscale thermophoresis for Agr versus PGC-1α. Agr, agrimol B; PGC-1α, peroxisome proliferator-activated receptor-γ coactivator 1α.

Molecular docking analysis was carried out to further explore the mechanism of Agr. The OH group of the benzene ring interacted with Agr at the GLU471 site *via* conventional hydrogen bonding, while the ketone group of the benzene ring and the methoxy group interacted with Agr at the LYS301 site *via* conventional hydrogen bonding. The modes of interaction of Agr and the PGC-1α protein are displayed in **Figure 2A**. The docking results revealed that the amino acid sites in PGC-1α are probably involved in binding with Agr, and the CDOCER energy was −40.9262 kcal/mol. The *K_D_* value of 2.4924 nM for the equilibrium dissociation constant was obtained by using more than 12 concentration gradients in MST (**Figure 2B**) and indicated the high binding affinity of Agr for PGC-1α. PGC-1α is an essential transcription co-factor for regulating cellular metabolism. Thus, Agr might play a role in cellular transcription by binding to PGC-1α, which can lead to mitochondrial biogenesis and promote the invasion and metastasis of cancer cells.

Proliferator-activated receptor-γ coactivator 1 (PGC-1) is a peroxisome proliferator-activated receptor PPARγ transcriptional coactivator. The CDOCER energy of Agr docking with the structure of PPARγ protein in complex with PGC-1α peptide was −35.8684 kcal/mol (**Supplementary Figure 1A**). To understand the potential combining capacity of PPARγ on Agr, the molecular docking assay was performed. The result showed that the CDOCER energy of Agr binding with PPARγ was −27.346 kcal/mol (**Supplementary Figure 1B**). This value was larger than the CDOCER energy of Agr binding with PCG-1α, which suggested that PCG-1α binding is stronger with Agr than PPARγ. In conclusion, PGC-1α may be the target protein of Agr.

### Agr suppressed mitochondrial activity of colon cancer cells *in vitro*

The MTT assay was performed using different concentrations of Agr (0, 36, 72, 144, 288, 576, and 1,152 nM). Agr dramatically inhibited HCT116 cell proliferation in a concentration-dependent manner. The half maximal growth inhibitory concentration (IC_50_) value was calculated as 280 nM using the equation shown in **Figure 1A**, and 144, 288, and 576 nM Agr were selected as test concentrations for subsequent experiments.

Next, we investigated the effect of Agr on the mitochondrial function of HCT116 cells by Hoechst 33342 staining and MitoTracker Red CMXRos staining. As shown in **Figures 1B, C**, 144, 288, and 576 nM Agr reduced the immunofluorescence intensity of MitoTracker compared with that of the control cells, suggesting that Agr inhibited mitochondrial activity in a concentration-dependent manner in HCT116 cells.

### Agr suppressed the effects of migration and proliferation of colon cancer cells *in vitro*

The mitochondrial activity serves as an important indicator for the migration and proliferation of cancer cells. As cancer cell migration is an essential element of tumor metastasis, we investigated the effects of Agr on HCT116 cell migration in a wound-healing assay. The cell migration rate was quantified by calculating the scratch area between moving cells at 0, 24, and 48 h using ImageJ software. Agr treatment significantly prevented HCT116 cell migration in a dose-dependent manner as shown in **Figures 1D, E**. Furthermore, we observed that the detectable fluorescence intensity was reduced in Agr-treated cells compared with that of the control cells, indicating that Agr inhibited the proliferation of HCT116 cells (**Figures 1F, G**). These results showed that Agr exerted tumor-suppressive activity toward CRC.

### Agr induced apoptosis of colon cancer cells *in vitro*

**Figure 3:**
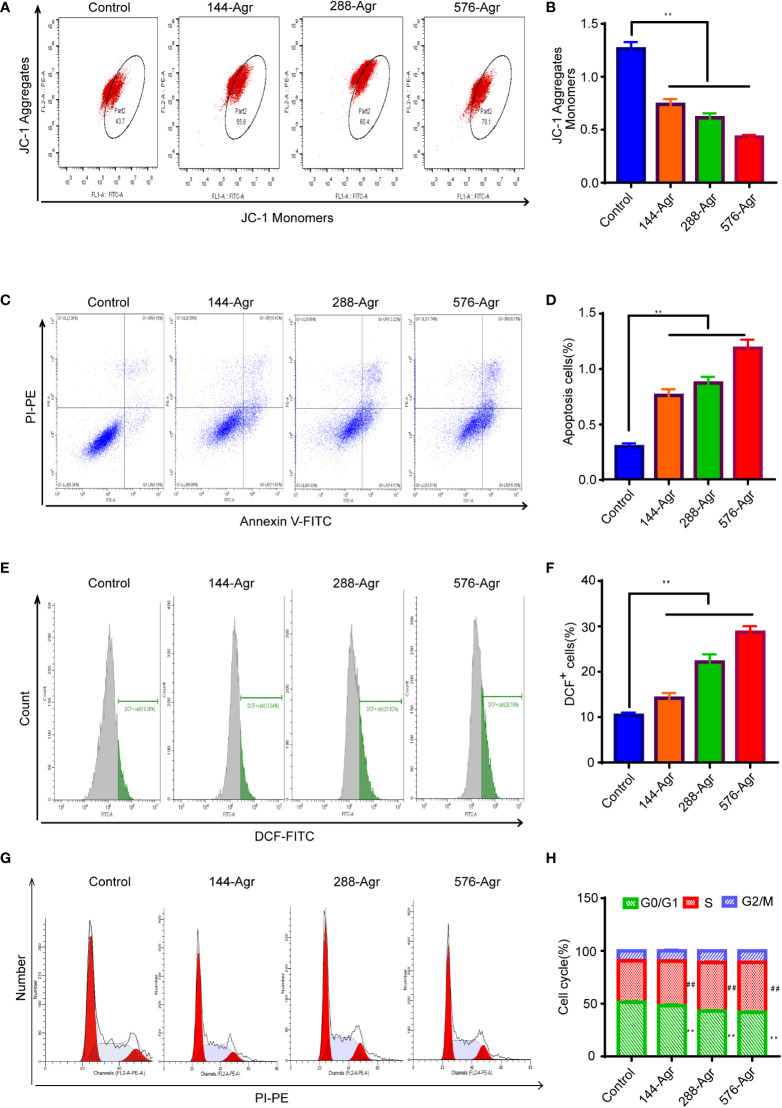
Agr induces cell apoptosis of HCT116 cells *in vitro*. **(A)** Effects of treatment with Agr on the mitochondrial membrane potential for CRC cells detected by JC-1 staining. **(B)** The ratio of (JC-1 aggregate)/(JC-1 monomer) fluorescence intensity shows unusual variations in mitochondrial membrane potential. A decrease in mitochondrial membrane potential indicates an increase in cell apoptosis. **(C)** After different concentrations (0, 144, 288, and 576 nM) of Agr, HCT116 cell apoptosis was determined by Annexin V–FITC/PI staining. **(D)** Quantitative data of Annexin V–FITC/PI staining assay; apoptosis of HCT116 cells after treatment with Agr. **(E)** Effect of Agr on ROS measured *via* the DCFDA assay for HCT116 cells. **(F)** Quantitative data of Agr on ROS production in HCT116 cells. **(G)** Quantitative data from the Annexin V–FITC/PI staining assay; apoptosis of HCT116 cells following treatment with Agr. **(H)** Cell cycle phase was detected after HCT116 cells were treated with Agr. Red zone (S phase cells), **p < 0.01 versus control. Green zone (G0/G1 cells), ^##^ *p* < 0.01 versus control. Agr, agrimol B; CRC, colorectal cancer; FITC, fluorescein isothiocyanate; PI, propidium iodide; ROS, reactive oxygen species.

Next, we explored the mechanism of the cell apoptosis induced by Agr. As apoptosis is known to involve the reduction in mitochondrial membrane potential, we examined the effect of Agr on mitochondrial membrane potential *via* JC-1 staining. Mitochondrial membrane potential was notably reduced in the Agr-treated cells compared to the control cells, as shown in **Figures 3A, B**. Moreover, **Figures 3C, D** show a significant decrease in Agr-treated cells compared to the control group.

Intracellular ROS accumulation induces oxidative stress, resulting in mitochondrial dysfunction and apoptosis of cancer cells (16). Hence, to further explore the apoptotic effect of Agr on HCT116 cells, we investigated ROS generation using the DCFDA-ROS fluorescent assay following the treatment of the cells with Agr. These experiments indicated that ROS generation rose significantly in Agr-treated cells compared to the control cells (**Figures 3E, F**), suggesting that Agr might induce cell apoptosis by elevating intracellular ROS levels. Additionally, Agr influenced the cell cycle distribution of CRC cells by increasing the non-proliferating cell fraction (G0/G1 phase) and decreasing the proliferating cell fraction (S phase) compared to the control cells (**Figures 3G, H**), suggesting that Agr might suppress tumorigenesis by affecting cell cycle progression.

### Agr decreased NRF1 and TFAM expression by binding to PGC-1α of colon cancer cells *in vitro*

**Figure 4:**
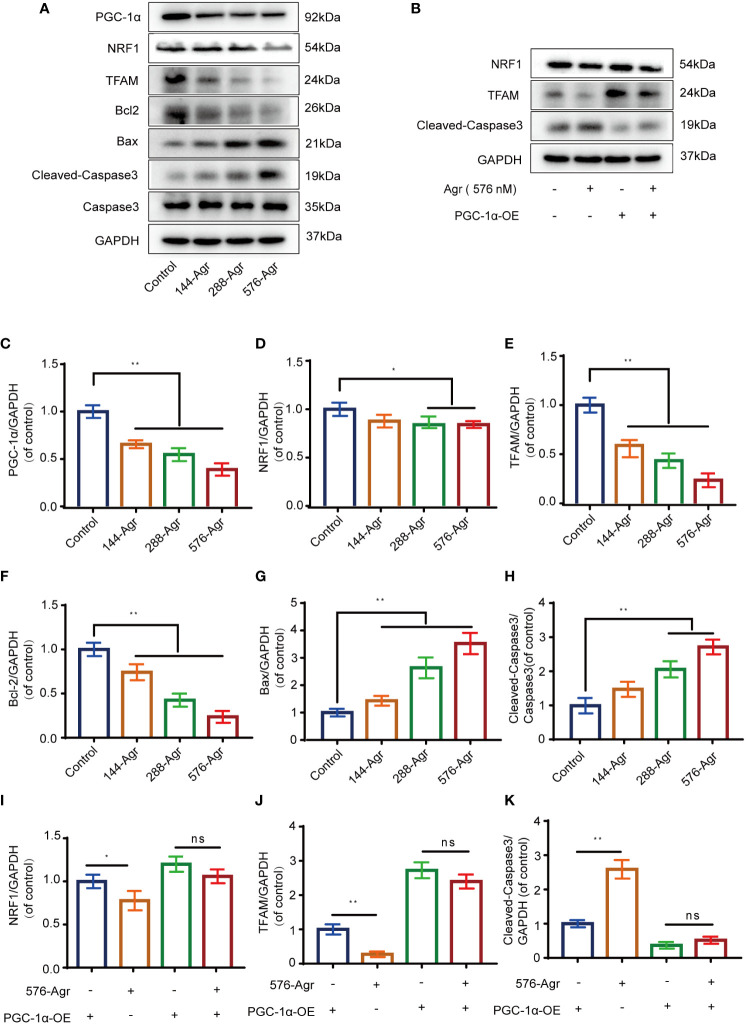
Agr reduces PGC-1α activation and NRF1 and TFAM expression of HCT116 cells *in vitro*. **(A)** After HCT116 cells were treated with different doses of Agr (0, 144, 288, and 576 nM) for 48 h, the protein levels of PGC-1α, NRF1, TFAM, Bax, Caspase-3, Bcl-2, and Cleaved-Caspase-3 were analyzed using Western blotting analysis. **(B)** After preliminary treatment of HCT116 cells with PGC-1α-OE for 30 min and treatment with 576 nM Agr for 48 h, the protein levels of NRF1, TFAM, and Cleaved-Caspase-3 protein were analyzed by Western blotting analysis. **(C)** Quantitative data for the ratio of PGC-1α to internal control protein (GAPDH). **p < 0.01 versus control. **(D)** Quantitative data for the ratio of NRF1 to GAPDH. *p < 0.05 compared with the control. **(E)** Quantitative data for the ratio of TFAM to GAPDH. **p < 0.01 compared with the control. **(F)** Quantitative data for the ratio of antiapoptotic proteins (Bcl-2) to internal control protein (GAPDH). **p < 0.01 versus control. **(G)** Quantitative data for the ratio of proapoptotic proteins (Bax) to internal control protein (GAPDH). **p < 0.01 versus control. **(H)** Quantitative data for the ratio of Caspase-3 to Cleaved-Caspase-3. **p < 0.01 versus control. **(I)** The protein levels of NRF1 were quantified by ImageJ. *p < 0.05, ns = no significant difference compared with the control. **(J)** The protein levels of TFAM were quantified using ImageJ. *p < 0.05, ns = no significant difference. **(K)** The protein levels of Cleaved-Caspase-3 were quantified using ImageJ. *p < 0.05, ns = no significant difference. The results are shown as mean ± SD (*n* = 3, each group). Agr, agrimol B; PGC-1α, peroxisome proliferator-activated receptor-γ coactivator 1α; NRF1, nuclear respiratory factor 1; TFAM, mitochondria transcription factor A.

The PGC-1α/NRF1/TFAM signaling pathway plays a crucial role in the mitochondrial pathway of apoptosis and mitochondrial biogenesis (17). Therefore, we analyzed the protein levels of PGC-1α, NRF1, and TFAM after treatment of HCT116 cells with Agr (144, 288, and 576 nM). Our preliminary results suggested that Agr significantly decreased the protein expression of PGC-1α, NRF1, and TFAM in HCT116 cells in a concentration-dependent manner (**Figures 4A, C–E**). We also found that Agr significantly increased protein levels of Bax and Caspase-3 and significantly decreased protein levels of Bcl-2 (**Figures 4A, F–H**). Taken together, these results led us to speculate that Agr-induced PGC-1α receptor inactivation resulted in the apoptosis of HCT116 cells.

To confirm the above results, an adenovirus vector (PGC-1α-OE) was used to overexpress PGC-1α in HCT116 cells. The overexpression of PGC-1α was found to increase the protein levels of NRF1 and TFAM and decrease that of Cleaved-Caspase-3. Treatment of PGC-1α-activated HCT116 cells with Agr (576 nM) decreased the protein levels of the above-mentioned PGC-α-related proteins (NRF1 and TFAM) and increased Cleaved-Caspase-3 levels (**Figures 4B, I–K**).

**Figure 5:**
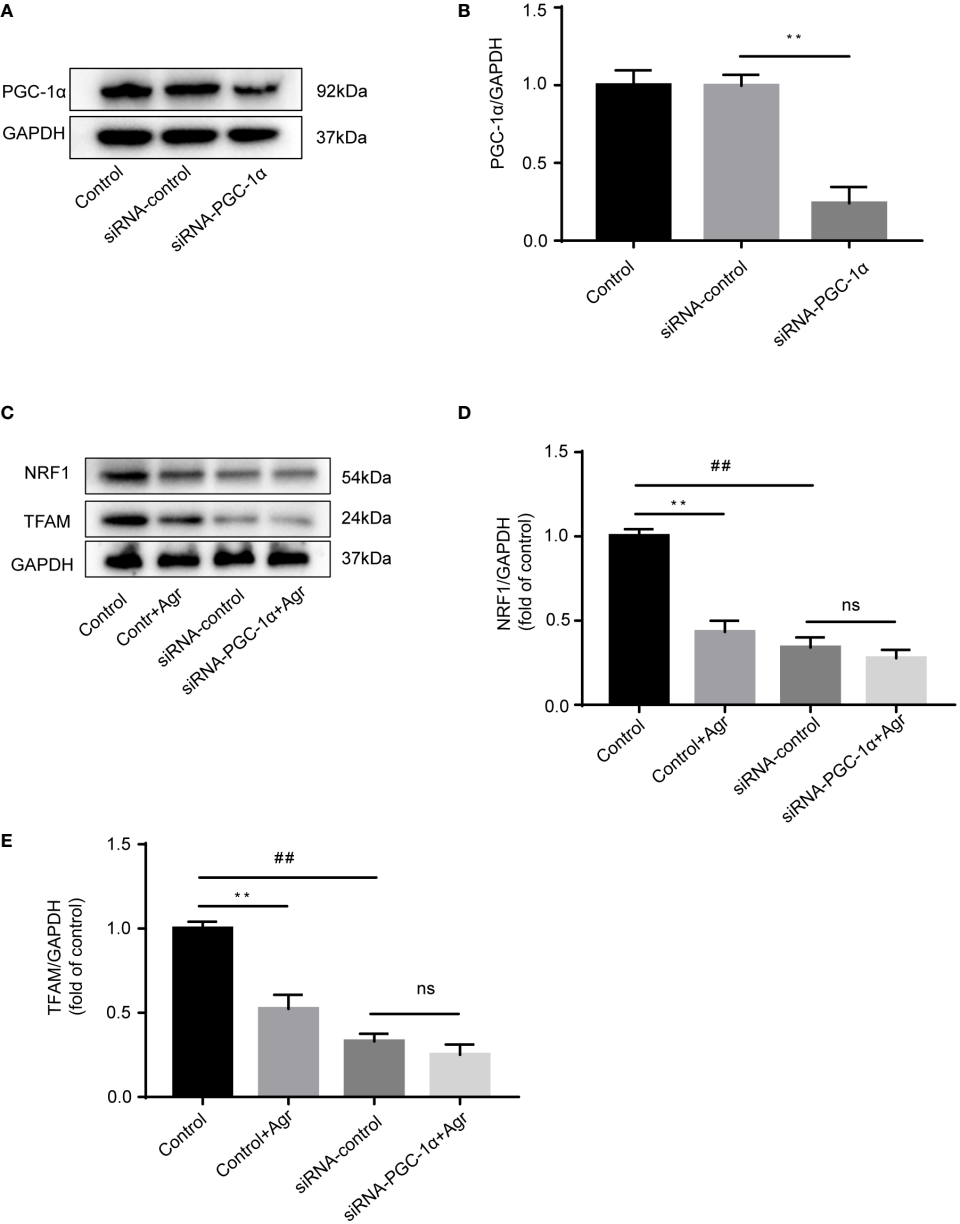
*PGC-1α* knockdown to show the effects of Agr on NRF1 and TFAM expression in HCT116 cells. **(A)** After HCT116 cells were transfected with adenovirus vector-based siRNA targeting the *PGC-1α* gene, PGC-1α protein levels were analyzed using Western blotting analysis. **(B)** Quantitative data for the ratios of PGC-1α to internal control protein (GAPDH). **p < 0.01, siRNA control versus siRNA-PGC-1α. **(C)** After HCT116 cells (siRNA-PGC-1α) were treated with Agr (576 nM), protein levels of NRF1 and TFAM were analyzed using Western blotting analysis. **(D)** The protein levels of NRF1 were quantified using ImageJ. **(E)** The protein levels of TFAM were quantified using ImageJ. **p < 0.01, control versus control-Agr. ^##^ *p* < 0.01, control-Agr versus siRNA-control. ns = no significant difference, siRNA-control versus siRNA-PGC-1α + Agr. The results are shown as mean ± SD (*n* = 3, each group). PGC-1α, peroxisome proliferator-activated receptor-γ coactivator 1α; Agr, agrimol B; NRF1, nuclear respiratory factor 1; TFAM, mitochondria transcription factor A.

Next, we knocked down *PGC-1α* to examine the effects of Agr on NRF1 and TFAM protein levels in HCT116 cells. Transfection of the siRNA targeting the *PGC-1α* gene into HCT116 cells resulted in decreased levels of the PCG-1α protein compared to the siRNA-control cells (**Figures 5A, B**). There was no difference in the protein levels of NRF1 and TFAM (**Figures 5C–E**) after treatment with Agr (576 nM) of HCT116 cells in the siRNA-PGC-1α cells compared with the siRNA-control cells. These data demonstrate that Agr-induced PGC-1α receptor inactivation promotes the apoptosis of colon cancer cells *in vitro*.

### Agr decreased NRF1 and TFAM expression and inhibited the progression of colon cancer *in vivo*

**Figure 6:**
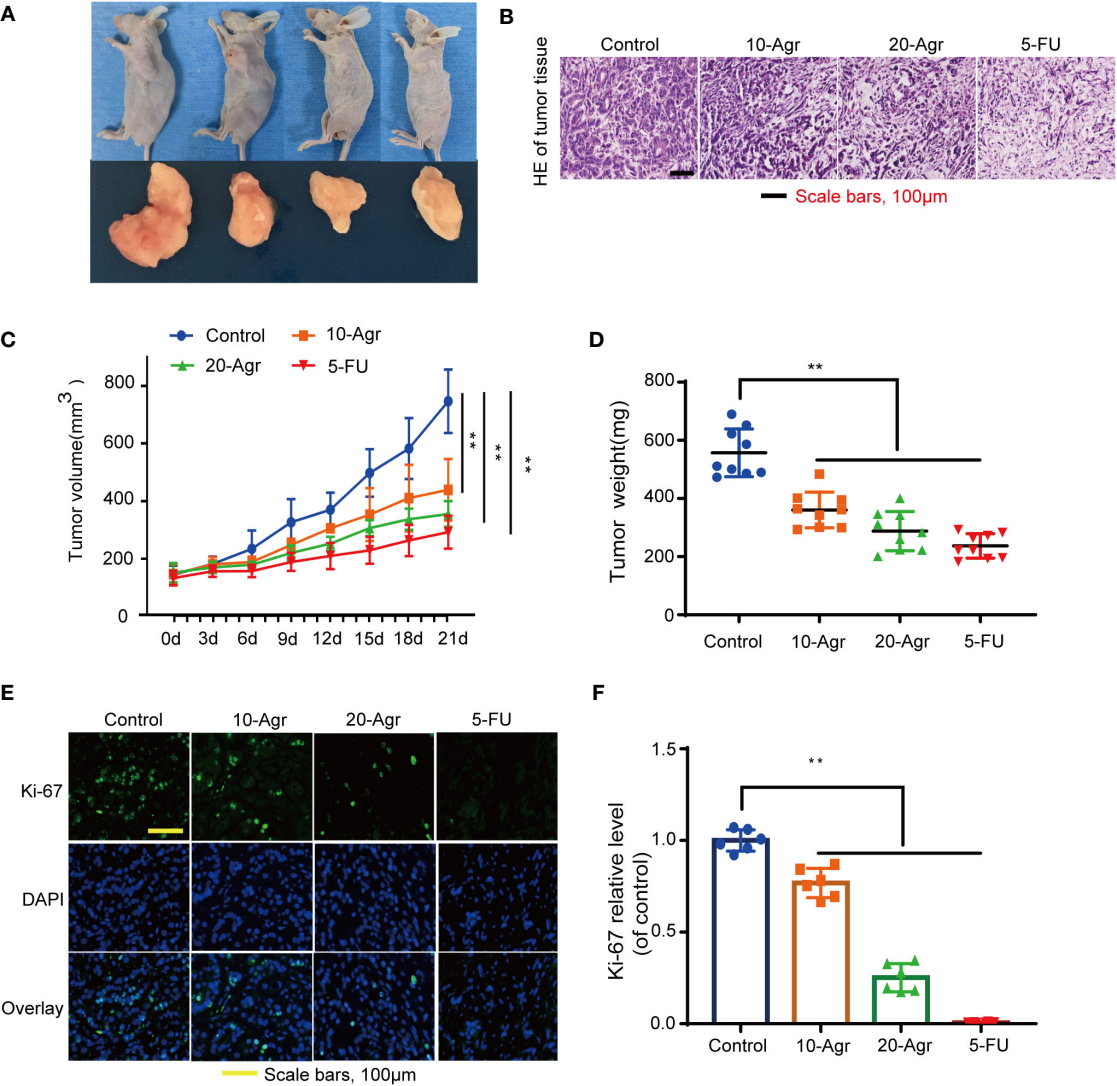
Agr inhibited the progression of colon cancer *in vivo*. 10-Agr represents Agr 10 mg/kg (i.p., QD), 20-Agr represents Agr 20 mg/kg (i.p., QD), and 5-FU represents 5-FU 5 mg/kg (i.p., QD). **(A)** A subcutaneous tumor model shows smaller tumors in Agr-treated groups than in the control group (*n* = 9, each group). **(B)** Representative micrographs showing tumor tissue by hematoxylin and eosin staining. **(C)** The volume of subcutaneous tumors following abdominal administration of drug (*n* = 9, each group). **(D)** Quantitative data of tumor weight (*n* = 9, each group). **(E)** Representative micrographs showing tumor tissue by Ki-67 fluorescent staining. **(F)** Quantitative data of Ki-67 fluorescent staining (*n* = 6). **p < 0.01 versus control. Scale bars = 100 μm. Agr, agrimol B.

Based on the previous results showing that Agr could promote apoptosis in CRC cells, we hypothesized that Agr would also suppress tumor growth by blocking PGC-1α/NRF1/TFAM signaling *in vivo*. HCT116 cells were subcutaneously injected into 35-day-old, male nude mice to establish a subcutaneous xenograft model, and the mice were treated with different concentrations of drugs (5 mg/kg 5-FU [positive control] and 10 and 20 mg/kg of Agr). As shown in **Figures 6A, C, D**, the volume and the weight of the tumors were significantly reduced in the Agr-treated groups compared with the other groups. Body weights and survival rates of the subcutaneously xenografted mice are presented in **Supplementary Figure 1**.

**Figure 7:**
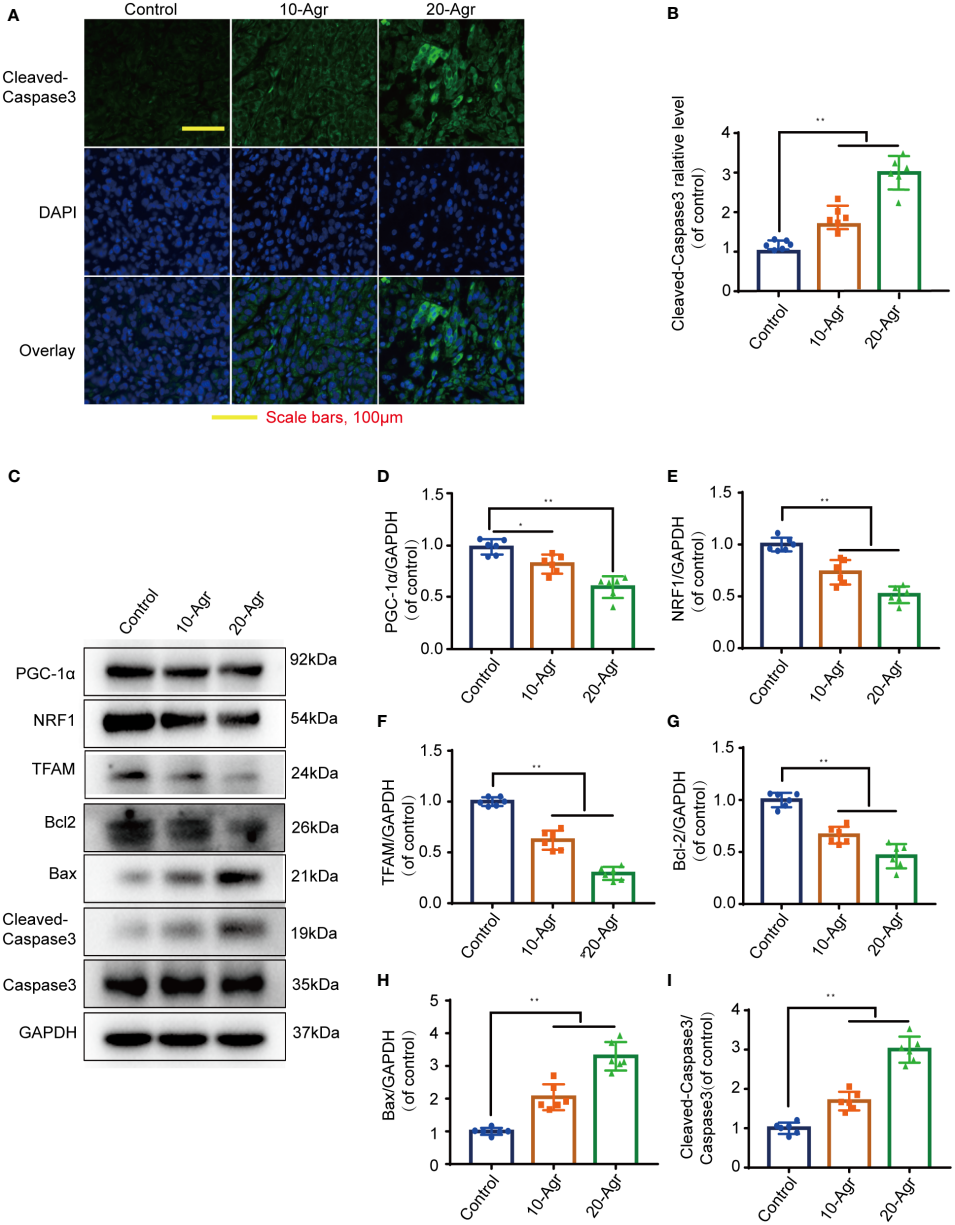
Agr decreased PGC-1α activation and NRF1 and TFAM expression in tumor tissue. 10-Agr represents Agr 10 mg/kg (i.p., QD), 20-Agr represents Agr 20 mg/kg (i.p., QD), and 5-FU represents 5-FU 5 mg/kg (i.p., QD). **(A)** Confocal fluorescence images of tumor tissue with Cleaved-Caspase-3 staining. Nuclei in blue (DAPI); antibody protein in green (Cleaved-Caspase-3). Scale bars, 100 μm. **(B)** Quantitative data for the relative Cleaved-Caspase-3 levels in a confocal fluorescence assay (*n* = 6). **(C)** The levels of TFAM, PGC-1α, NRF1, Cleaved-Caspase-3, Bcl-2, Caspase-3, and Bax were analyzed in the tumor tissue by Western blotting analysis (*n* = 3). **(D–H)** Quantitative data for the ratios of PGC-1α, NRF1, TFAM, Bcl-2, and Bax to internal control protein (GAPDH) in tumor tissue. **(I)** Quantitative data for the ratio of Cleaved-Caspase-3 to Caspase-3. *p < 0.05, **p < 0.01 versus control. Scale bars = 100 μm. Agr, agrimol B; PGC-1α, peroxisome proliferator-activated receptor-γ coactivator 1α; NRF1, nuclear respiratory factor 1; TFAM, mitochondria transcription factor A.

The pathological features of the tumor samples were examined by H&E staining. As shown in **Figure 6B**, the infiltration of inflammatory cells into tumor tissue of Agr-treated group mice was decreased in comparison with that of the control group. Furthermore, the relative expression levels of proliferating cell nuclear antigen (Ki-67) and PCNA were increased in the tumor samples of Agr-treated groups compared with those of the control groups (**Figures 6E, F**). Agr treatment increased the activity of Cleaved-Caspase-3, as evidenced by immunofluorescence analysis (**Figures 7A, B**). Agr treatment decreased TFAM, NRF1, PGC-1α, and Bcl-2 protein levels but elevated the protein levels of Cleaved-Caspase-3 and Bax (**Figures 7C-I**). Thus, these results indicate that Agr could regulate tumor proliferation and inhibit tumor growth by interfering with the activity of PGC-1α and related proteins *in vivo*, which was consistent with the results of the *in vitro* experiments.

## Discussion

Sustaining proliferative signaling and resisting cell death are important hallmarks of cancer (18). The rapid proliferation of cancer cells is an important cause of the reduced overall survival of CRC patients, and liver metastasis is the primary cause of high mortality (19). The fecal immunochemical and fecal occult blood tests are non-invasive screening tests that are suitable for people who cannot accept colonoscopy. The “seed and soil hypothesis” of cancer postulates that cancer cells need to absorb large amounts of nutrients from their specific environment to achieve metastatic outgrowth (20). Glucose is the main metabolic resource, which is completely converted to adenosine 5′-triphosphate (ATP) by mitochondrial oxidative phosphorylation and aerobic glycolysis. Tumorigenesis is an energy-dependent process that requires the breakdown and synthesis of ATP. Hence, energy metabolism pathways are highly attractive targets in tumor cells for achieving the goal of cancer prevention (21, 22).

Mitochondria are the main energy-producing organelles in human cells, providing energy for cancer cell transition, proliferation, infiltration, and metastasis. A sharp increase in energy metabolism is necessary for the rapid proliferation of cancer cells (23). Multiple mitochondrial biological processes, including mitochondrial biogenesis, mitochondrial oxidative stress, maintenance of mitochondrial DNA homeostasis, and mitophagy, affect the metabolic status of tumors (24–26). Tumor suppressors directly regulate mitochondrial biology (27). Thus, mitochondria have a dual role in cancer, which may open up new avenues for CRC therapy. We had previously reported that Agr induces mitochondrial dysfunction of CRC cells through the inhibition of mitochondrial biogenesis and oxidative stress.

Mitochondrial biogenesis—the process of impaired, old mitochondria to form new mitochondria *via* transcriptional programs—is an adaptive response to cellular stress and environmental stimuli. Mitochondrial biogenesis increases mitochondrial assembly and maintains mitochondrial function, thereby providing opportunities to disrupt mitochondrial homeostasis in pathophysiological cases of drug/toxicant exposure (28). Mitochondrial biogenesis is favored in a few highly aggressive cancer cells such as CRC and osteosarcoma. CRC induces mitochondrial biogenesis pathways by upregulating the expression of several transcription factors (PGC-1α, NRF1, and TFAM) and crucial enzymes. Lysine demethylase 3A inhibits *PGC-1α* monomethylation. PGC-1α induces NRF1-dependent transcriptional regulation of TFAM to regulate mitochondrial biogenesis (6). The mitochondrial calcium uniporter inhibits the phosphorylation of TFAM by controlling mitochondrial calcium levels, thus accelerating mitochondrial biogenesis in CRC *via* NF-κB signaling (24). In this study, Agr treatment inhibited CRC proliferation and migration by preventing mitochondrial biogenesis and oxidative stress *in vitro* and *in vivo.*

Mitochondrial oxidative stress is a metabolism disorder that is characterized by the excessive production of ROS resulting in damage to mitochondrial proteins and DNA. Mitochondria generate ATP by mitochondrial oxidative phosphorylation to provide energy for cellular functions. They also generate certain macromolecules, including fatty acids and cholesterol, which form lipid bilayer membranes for constant cell division. ROS comprises superoxide free radicals, hydroxyl free radicals, hydrogen peroxide, and other oxidation products of oxygen-containing substances by mitochondrial energy metabolism. In response to various stimuli, including senescence, chemical toxins, hypoxia, environmental pollution, and drugs, mitochondrial stresses promote excessive ROS production. ROS cleaves and degrades mitochondrial proteins, modulating mitochondrial DNA transcription, damaging mitochondrial integrity, enhancing cytochrome c release, and inducing intrinsic apoptosis initiation. The formation of dysfunctional mitochondria is reported to block the survival, proliferation, and migration of cancer cells (29). Our data indicate that Agr decreases mitochondrial membrane potential, contributing to the enhanced production of ROS and exacerbating mitochondrial oxidative stress and mitochondrial damage for CRC cells.

PGC-1α, a powerful transcription coregulator of mitochondrial genes, interacts with transcription factors (NRF1 and TFAM) and modulates multiple biological processes of mitochondrial metabolism, including mitochondrial biogenesis and mitochondrial oxidative stress (3). PGC-1α activates the promoter of *NRF1*, leading to its transcription. NRF1 is sensitive to free radical signaling and functions as an indicator of cellular redox status. NRF1 promotes the transcription and translation of *TFAM*, which is transported into mitochondria where it combines with mitochondrial DNA to activate RNA polymerase, which activates mitochondrial genome transcription. Rapamycin regulates mitochondrial biogenesis and mitochondrial energy production *via* PGC-1α/Yin Yang 1 activation. The upregulation of *PGC-1α* expression results in a protective effect on cardiac cells and nerve cells through the initiation and maintenance of mitochondrial biogenesis. However, growing evidence has revealed that PGC-1α mediates survival and proliferation in tumorigenesis, and the overexpression of *PGC-1α* is closely linked to enhanced mitochondrial biogenesis in CRC cells (30, 31) and suppression of cancer cell growth depending on the resistance of these cells to oxidative stress (5). Studies have shown that many polyphenolic compounds have a high binding affinity with PGC-1α (32, 33), while Agr has a similar polyphenolic structure. Therefore, we speculated that Agr might bind with PGC-1α. The molecular and microscale thermography results demonstrated that Agr could dock with PGC-1α. The present study demonstrates that PGC-1α is crucial for the proliferation and migration of HCT116 cancer cells, suggesting that maintaining PGC-1α levels might be a successful anticancer strategy. Our results showed that Agr may interact with PGC-1α and reduce the protein levels of NRF1 and TFAM, thereby promoting mitochondrial biogenesis.

Previous studies have shown that agrimophol could stimulate mitochondria-dependent apoptosis in cancer cells. Thus, Agr extracted from agrimophol also suppresses cell proliferation by enriching cells in the G phase (10, 12). In this study, Agr was found to increase ROS generation, block Bcl-2 expression, and increase Caspase-3 and Bax expression to promote cancer cell apoptosis.

## Conclusion

**Figure 8:**
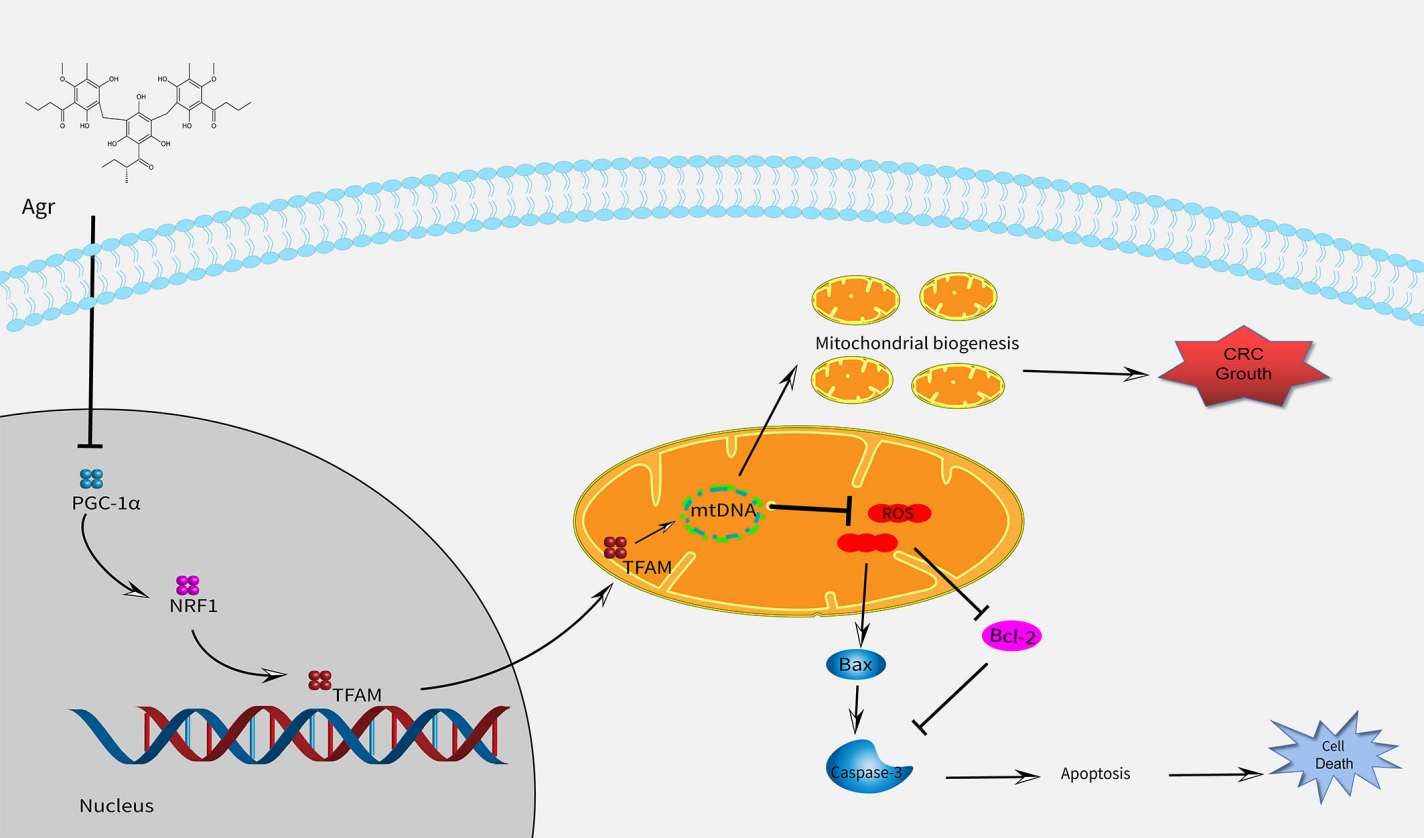
A schematic model showing that Agr alters mitochondrial biogenesis and can cause cell death by decreasing the expression of PGC-1α/NRF1/TFAM. Agr, agrimol (B); PGC-1α, peroxisome proliferator-activated receptor-γ coactivator 1α; NRF1, nuclear respiratory factor 1; TFAM, mitochondria transcription factor (A).

In conclusion, we found that Agr inhibited colon carcinoma progression by promoting oxidative stress and cell apoptosis by blocking the PGC-1α/NRF1/TFAM signaling pathway (**Figure 8**). Furthermore, *in vitro* and *in vivo* tests have shown that the HCT116 cell proliferation was inhibited after treatment with Agr, demonstrating the potential benefits of Agr for clinical use and paving the way for its development as a therapeutic agent for the treatment of CRC.

## Data availability statement

The original contributions presented in the study are included in the article/**Supplementary Material**. Further inquiries can be directed to the corresponding authors.

## Ethics statement

The animal experiment protocol complied with international guidelines and was also approved by the Nanjing University of Chinese Medicine (No. 202204A033).

## Author contributions

DX and WY were responsible for drafting the manuscript. JM, XP, QY and JT contributed to the analysis and interpretation of data. ZF, DT, LL, and CY contributed to the data collection. JQ, HC, and DS directed the project and wrote, reviewed, and edited the manuscript. All authors contributed to the article and approved the submitted version.
