# Supplementary figures and images for: Agrimol B inhibits colon carcinoma progression by blocking mitochondrial function through the PGC-1α/NRF1/TFAM signaling pathway
Source: Front Oncol. 2022 Dec 14;12:1055126. doi: 10.3389/fonc.2022.1055126 (PMC9794846; doi:10.3389/fonc.2022.1055126)

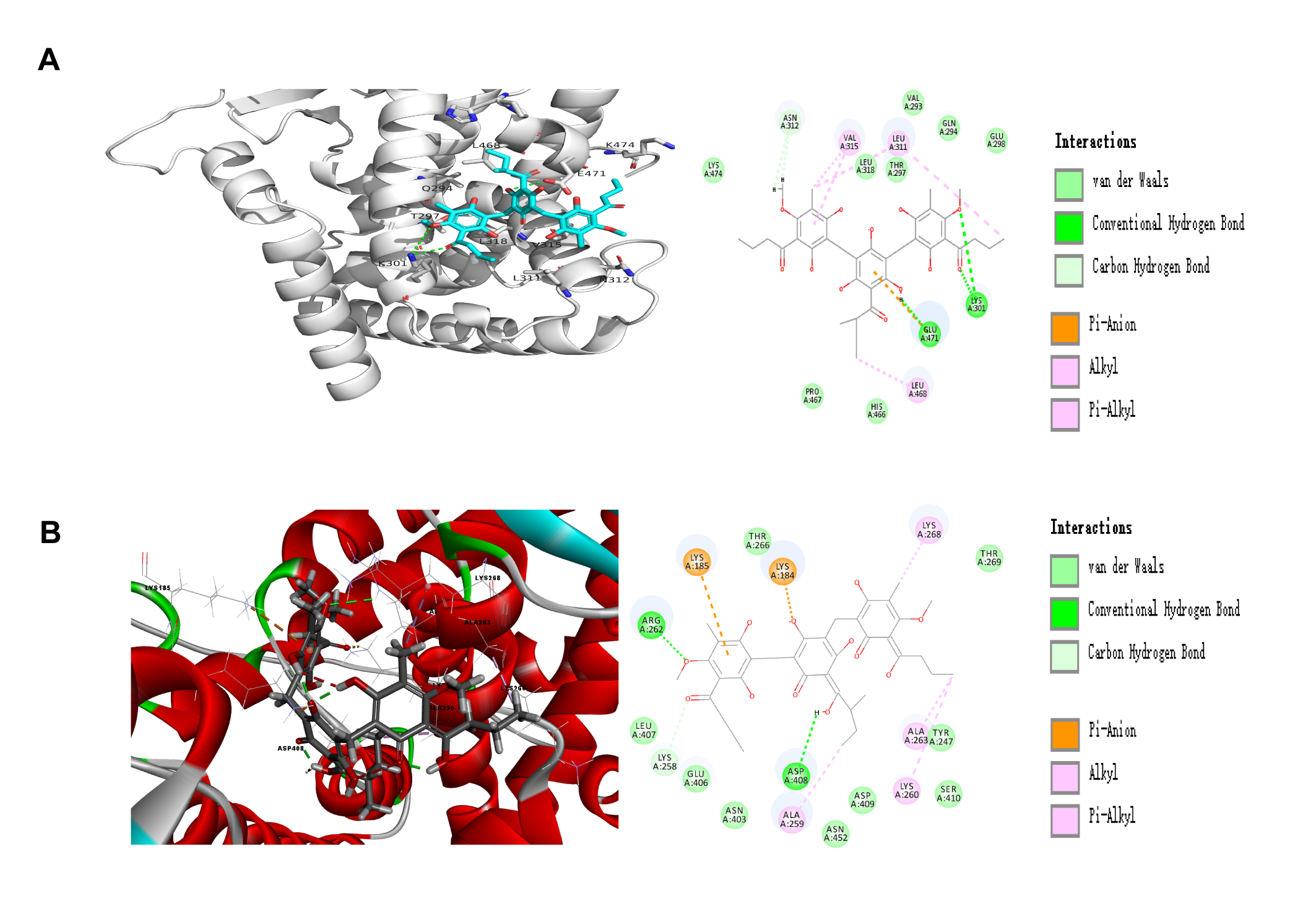

Supplement: Supplementary file 1 [file Image_1.tif]

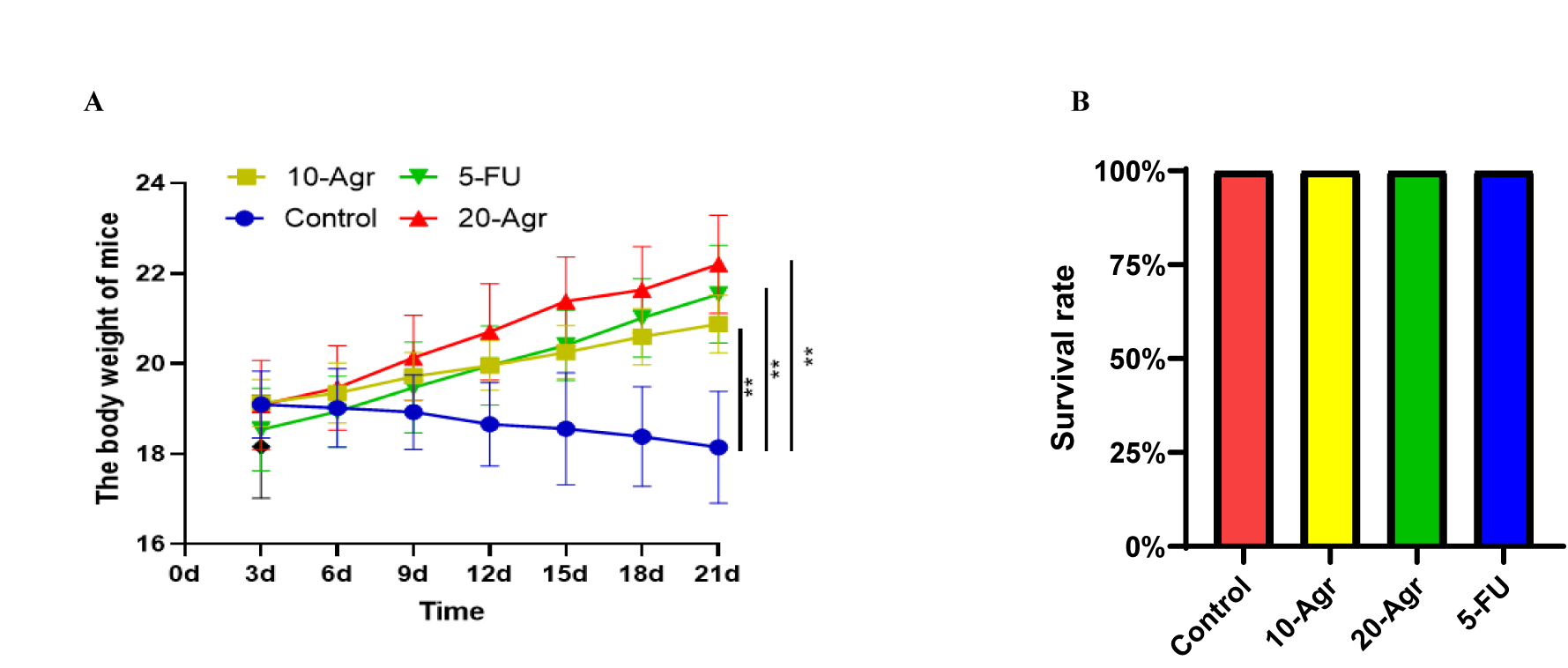

Supplement: Supplementary file 2 [file Image_2.tif]
